# Localization of Potential Energy in Hydrogen Bonds of the *ATXN2* Gene

**DOI:** 10.3390/ijms26030933

**Published:** 2025-01-23

**Authors:** Mikhail Drobotenko, Oksana Lyasota, Stepan Dzhimak, Alexandr Svidlov, Mikhail Baryshev, Olga Leontyeva, Anna Dorohova

**Affiliations:** 1 Research Department, Kuban State University, 350040 Krasnodar, Russia4098789@mail.ru (O.L.); elkina0131@gmail.com (A.D.); 2 Laboratory of Problems of Stable Isotope Spreading in Living Systems, Southern Scientific Center of the Russian Academy of Sciences, 344006 Rostov-on-Don, Russia; svidlov@mail.ru (A.S.);

**Keywords:** *ATXN2* gene, CAG tract, diseases of trinucleotide repeat expansion, DNA model, torque, viscosity, energy localization

## Abstract

It is known that a number of neurodegenerative diseases, also called diseases of waiting, are associated with the expansion of the polyQ tract in the first exon of the *ATXN2* gene. In the expanded polyQ tract, the probability of occurrence of non-canonical configurations (hairpins, G-quadruplexes, etc.) is significantly higher than in the normal one. Obviously, for their formation, the occurrence of open states (OSs) is necessary. Calculations were made for these processes using the angular mechanical model of DNA. It has been established that the probability of the large OS zones genesis in a DNA segment depends not only on the “strength” of the nucleotide sequence but also on the factors determining the dynamics of DNA; localization of the energy in the DNA molecule and the potential energy of interaction between pairs of nitrogenous bases also depend on environmental parameters. The potential energy of hydrogen bonds does not remain constant, and oscillatory movements lead to its redistribution and localization. In this case, OSs effectively dissipate the energy of oscillations. Thus, mathematical modeling makes it possible to calculate the localization of mechanical energy, which is necessary for the OSs formation, and to predict the places of their origin, taking into account the mechanical oscillations of the DNA molecule.

## 1. Introduction

The first exon of the *ATXN2* gene contains a site of tandemly repeated CAG sequences that form a polyglutamine (polyQ) tract [1,2,3,4]. In a healthy man, the CAG repeat tract in the *ATXN2* gene consists of approximately 22–23 CAG trinucleotides [5,6,7]. An increase in the number of CAG repeats can lead to several pathologies: ataxia, amyotrophic lateral sclerosis (ALS), and parkinsonism [7,8,9,10,11,12,13].

The repeat expansion associated with spinocerebellar ataxia type II (SCA2) is greater than 34 and typically represents pure CAG repeats [14,15,16,17,18]. Parkinsonism is associated with a CAG repeat region interrupted by CAA interruptions, compared with the pure CAG repeats typical for SCA2 [19]. Repeats associated with increased risk of ALS consist of shorter CAG sequences interrupted by 1–3 CAA interruptions [20,21,22]. Age at onset of ALS varies significantly depending on the number of CAA interruptions [21,23,24].

It is important to understand how the nucleotide sequence in the CAG repeat region affects the manifestation of pathology. The results of the studies allowed us to suggest that the sequence of repeats affects the stability of the tract [25,26,27]. Pure CAG tract is unstable and tends to expand [28,29,30,31]. It is difficult to detect the dependencies and patterns of such instability experimentally since this requires a large amount of experimental data. Recently, attempts have been made to solve this problem with the help of mouse models [32,33,34]. The most accessible method that allows conducting a large number of experiments and analyzing different options is mathematical modeling. Full-atom modeling in studying the conformational and dynamic properties of nucleic acids has a number of limitations. It requires large computational costs; the study can only be carried out on short time intervals, within which only short nucleotide sequences can be studied. Mechanical models of DNA are currently very popular, they use a number of simplifications but allow studying long sequences over fairly large time intervals [35,36,37,38,39].

The fact that CAA interruptions and continuous CAG sequences of the same length in the *ATXN2* gene lead to different diseases: SCA2 or parkinsonism, confirms that sequence configuration and repeat length are important factors in the clinical manifestations and phenotypic variability of the *ATXN2* gene [40,41,42,43].

It has been established that the presence of CAA interruptions in the right part of the CAG tract with a high probability leads to the genesis of an additional open states (OS) zone. The calculations made allowed us to establish that CAA interruptions affect the stability of the CAG tract. This influence, depending on the localization of the interruption, can either increase the stability of the CAG tract or decrease it [44]. An open state is a base pair with broken hydrogen bonds between complementary nucleotides.

Toxicity and cell death in SCA2 are thought to be related to the formation of pathogenic secondary structures in DNA, such as hairpins, Z-DNA, triple helices, G-quadruplexes, and various slip strand duplexes [6,45,46,47,48,49,50,51]. It is very important to understand their structural and dynamic characteristics since they can trigger a chain of molecular mechanisms that lead to the development of pathology [52,53].

Another approach relates the development of pathologies with the stability of the CAG tract. Using mathematical modeling methods, it was shown that with an increase in the length of the CAG tract, the probability of the appearance of additional large open state (OS) zones in it increases. A correlation was established between this probability and statistical data on the age of onset of the disease [54]. It is obvious that for the emergence of secondary structures (hairpins, G-quadruplexes, etc.), a necessary condition is the appearance of large OS zones in the CAG tract.

Calculations performed using the mechanical model allowed us to establish the following patterns: an increase in the length of the CAG tract (>40 CAG repeats) or a decrease in the viscosity of the medium surrounding the DNA molecule leads to a decrease in the stability of the CAG tract; and an increase in the viscosity of the CAG tract leads to stabilization of the DNA molecule (a decrease in the probability of the of OS zones genesis). In addition, in the zone close to incomplete penetrance of the disease, viscosity does not have a reliable effect on the stability of the CAG tract [55]. Previous studies have shown that additional OS zones arise precisely in the CAG tract, in which AT pairs make up only one-third of the part. At the same time, near the CAG tract, there are zones with a high content of AT pairs. To explain this fact, it is necessary to take into account the influence of oscillation movements of the DNA molecule on the formation of open states [56].

To take this influence into account, in this work, we investigated the dynamics of the potential energy of hydrogen bonds in base pairs and its influence on the occurrence of OSs. The *ATXN2* gene with low stability of the CAG tract containing 50 CAG repeats was selected for the study. It was found that DNA oscillations lead to the redistribution of the potential energy of hydrogen bonds and their concentration in the segment of the additional OS zones genesis in the CAG tract. The influence of external parameters, such as force effect and viscosity of the surrounding liquid, on the distribution of potential energy of hydrogen bonds, was studied. In addition, the influence of CAA interruptions in the CAG tract on the genesis of OS zones and the distribution of hydrogen bonds’ potential energy in base pairs was established.

## 2. Results and Discussion

Calculations were performed for the *ATXN2* gene, containing 50 CAG repeats with torque localized in the segment [5233, 5900]. The beginning of the localization segment coincides with the beginning of the promoter, the end of the segment was chosen based on the results obtained in the work [54].

Calculations were carried out on the gene segment from the 4601st to 6681st base pairs of the *ATXN2* gene. This was undertaken to reduce the program execution time, since for the purposes of this study, the segment must contain the first exon of the gene and the CAG tract. In addition, the area from the 4601st to 6681st base pairs was selected taking into account the fact that under the external influence (torque), the disturbance zone does not extend beyond the boundaries of the selected area. This allows us to set the boundary conditions adequately for the calculated time interval.

**Figure 1:**
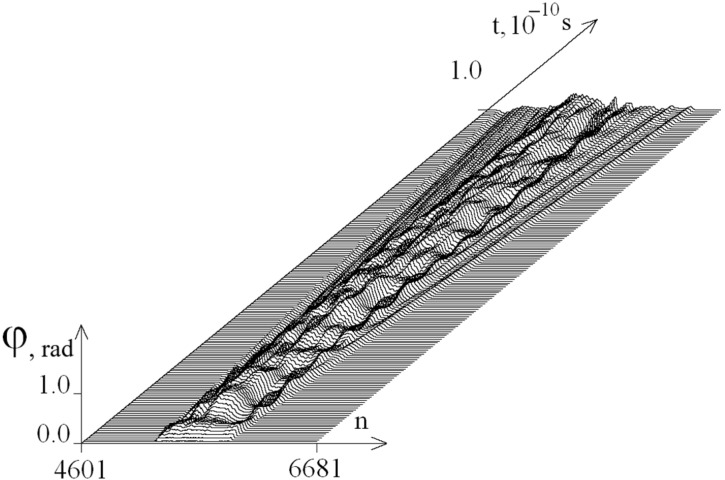
Dynamics of angular deviations of the first chain of the selected DNA fragment over the time interval [0; 10^−10^ s] with a torque M_0_ = 8.00 pN·nm and a viscosity parameter of the medium λ = 1.

The calculation results are presented in the form of figures. Figure 1 shows the graphs of angular deviations of the first chain of the selected DNA fragment over the time interval [0; 10^−10^ s] with the magnitude of the torque M_0_ = 8.00 pN·nm and the viscosity parameter of the medium λ = 1. It is evident that the disturbance caused by the torque does not reach the boundaries of the selected region of the gene.

### 2.1. Distribution of Potential Energy of Hydrogen Bonds with a Change in the Magnitude of the Torque M_0_

**Figure 2:**
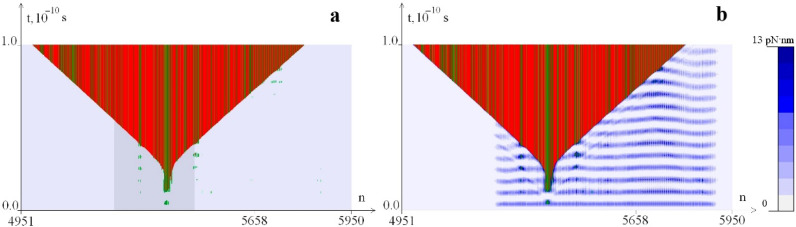
Dynamics of OS zones genesis and potential energy of hydrogen bonds in base pairs at M_0_ = 8.28 pN·nm. OS in AT pairs is shown in green, and OS in GC pairs is shown in red, and the promoter is highlighted with a darker background (**a**). Closed base pairs are highlighted in blue depending on the potential energy values of hydrogen bonds in accordance with the color scale shown on the right (**b**).

For λ = 1, the OS zones in the promoter region appear at the torque value M_0_ > 8.28 pN·nm. Figure 2 shows the dynamics of the OS zones genesis and the potential energy of hydrogen bonds in base pairs at M_0_ = 8.28 pN·nm. The numbers of nitrogenous base pairs are marked horizontally, and time is shown vertically. The OS in AT pairs is shown in green in the figure, and the OS in GC pairs is shown in red. The promoter is highlighted with a darker background; the beginning of the CAG tract is indicated (the 5658th base pair). Closed base pairs are highlighted in blue depending on the potential energy of hydrogen bonds; the lightest shade corresponds to pairs with a potential energy of <1.00 pN·nm, and the darkest—to pairs with a potential energy of >12.00 pN·nm.

From Figure 2b, it is clear that the distribution of the potential energy of hydrogen bonds depends on time: at first, the potential energy is evenly distributed between base pairs, and then it is redistributed and localized on individual DNA sections.

**Figure 3:**
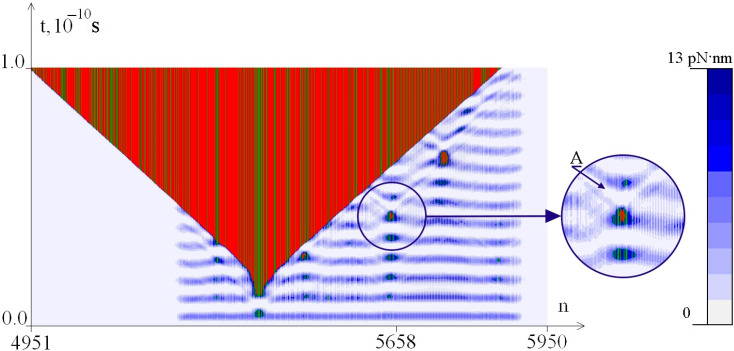
Dynamics of OS zones genesis and potential energy of hydrogen bonds in base pairs at M_0_ = 8.49 pN·nm. The green color in the figure indicates the OS in AT pairs, and the red color indicates the OS in GC pairs. Closed base pairs are highlighted in blue depending on the potential energy values of hydrogen bonds in accordance with the color scale shown on the right.

**Figure 4:**
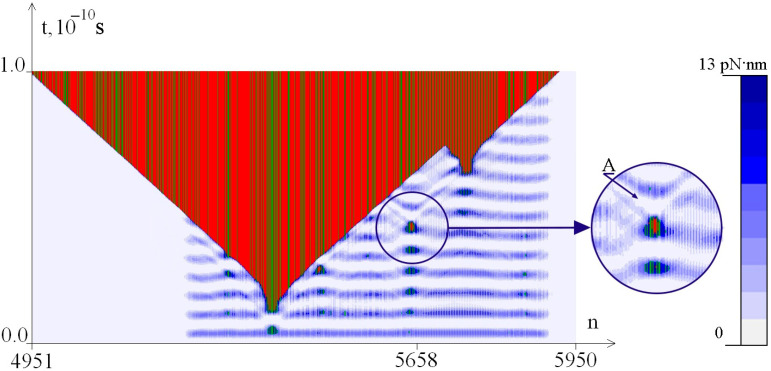
Dynamics of OS zones genesis and potential energy of hydrogen bonds in base pairs at M_0_ = 8.50 pN·nm. OS in AT pairs is shown in green, and OS in GC pairs is shown in red. Closed base pairs are highlighted in blue depending on the potential energy values of hydrogen bonds in accordance with the color scale shown on the right.

**Figure 5:**
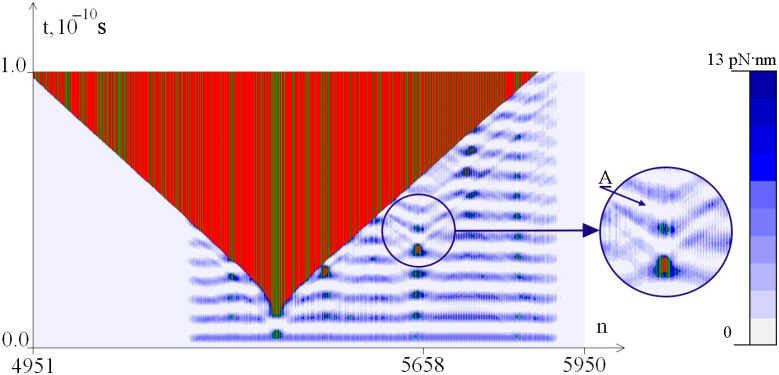
Dynamics of OS zones genesis and potential energy of hydrogen bonds in base pairs at M_0_ = 8.55 pN·nm. OS in AT pairs is shown in green, and OS in GC pairs is shown in red. Closed base pairs are highlighted in blue depending on the potential energy values of hydrogen bonds in accordance with the color scale shown on the right.

Figure 3, Figure 4 and Figure 5 show the dynamics of the OS zones genesis and the distribution of the potential energy of hydrogen bonds in base pairs depending on the value of M_0_. It can be seen that the potential energy of hydrogen bonds is localized in the CAG tract, which can lead to the appearance of additional OS zones of various sizes.

Thus, at M_0_ = 8.49 pN·nm (Figure 3), the accumulated energy is only sufficient to occur in a small OS zone, while at M_0_ = 8.50 pN·nm (Figure 4), we see the large OS zone genesis.

Enlarged fragments of the figures show that the appearance of small OS zones leads to a local decrease in the potential energy of hydrogen bonds in the opening areas. This can be explained by an increase in the angular velocity of base pairs in the OS zones and, consequently, a more intense dissipation of oscillation energy due to interaction with the environment. Therefore, a stronger torque can lead to earlier localization of potential energy sufficient for the formation of a small OS zone, which, due to the more intense dissipation of oscillation energy, will not allow the further formation of a large OS zone. This explains the non-monotonicity of the formation of OS zones with an increase in the magnitude of the torque M_0_, which can be seen when comparing Figure 4 (M_0_ = 8.50 pN·nm) and Figure 5 (M_0_ = 8.55 pN·nm). This non-monotonicity has been systematically identified in the works [57,58].

The enlarged fragment shows (Figure 3) that the appearance of a small OS zone in the area occurs with a local decrease in the potential energy of hydrogen bonds, indicated by arrow A.

Figure 4 shows how the localization of the potential energy of hydrogen bonds in the CAG tract led to the formation of a large additional OS zone. The enlarged fragment shows that in the area where the small OS zone appears, there is a local decrease in the potential energy of hydrogen bonds, indicated by arrow A.

The enlarged fragment shows (Figure 5) that in the area of the emergence of a small OS zone, there is a local decrease in the potential energy of hydrogen bonds, indicated by arrow A. This did not allow the formation of a large additional OS zone in the CAG tract.

Calculations show that, in general, an increase in the value of torque leads to an increase in the probability of the occurrence of additional large-sized OS zones [58]; however, this dependence is not monotonic.

### 2.2. The Influence of the Environment Viscosity on the Distribution of Hydrogen Bonds Potential Energy

**Figure 6:**
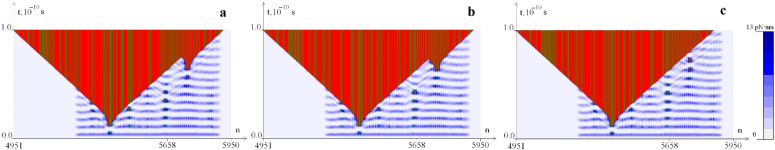
Dynamics of OS zones genesis and potential energy of hydrogen bonds in base pairs at M_0_ = 8.50 pN·nm for different values of the parameter characterizing the viscosity of the environment: λ = 0.9 (**a**), λ = 1.0 (**b**), and λ = 1.1 (**c**). The green color in the figure indicates the OS in AT pairs, and the red color indicates the OS in GC pairs. Closed base pairs are highlighted in blue depending on the potential energy values of hydrogen bonds in accordance with the color scale shown on the right.

Figure 6 shows the dynamics of OS zones genesis and the distribution of the potential energy of hydrogen bonds in base pairs at M_0_ = 8.50 pN·nm for different values of λ, which characterizes the viscosity of the environment. It is evident that at λ = 0.9 and λ = 1.0, additional large OS zones appear in the CAG tract, while at λ = 1.1, they do not; i.e., in this case, the increase in viscosity is a stabilizing factor.

**Figure 7:**
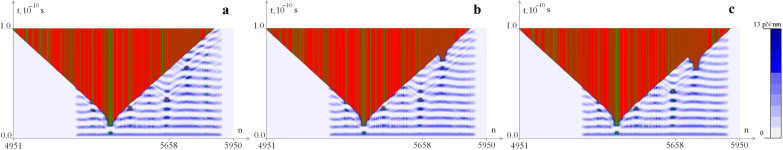
Dynamics of OS zones genesis and potential energy of hydrogen bonds in base pairs at M_0_ = 8.52 pN·nm for different values of the parameter characterizing the viscosity of the environment: λ = 0.9 (**a**), λ = 1.0 (**b**), and λ = 1.1 (**c**). The green color in the figure indicates the OS in AT pairs, and the red color indicates the OS in GC pairs. Closed base pairs are highlighted in blue depending on the potential energy values of hydrogen bonds in accordance with the color scale shown on the right.

Calculations show that, in general, an increase in viscosity leads to a decrease in the probability of the occurrence of additional large OS zones [57]; however, this dependence is not monotonic. This is evident from Figure 7, which shows the dynamics of OS zones genesis and the distribution of the potential energy of hydrogen bonds in base pairs at M_0_ = 8.52 pN·nm, depending on the value of λ, which characterizes the viscosity of the environment. In this case, at λ = 0.9, small OS zones genesis in the CAG tract, and additional large OS zones genesis at λ = 1.0 and λ = 1.1. This is explained by the fact that the process of redistribution of the potential energy of hydrogen bonds in base pairs and localization of potential energy sufficient for the formation of OS zones is nonlinear.

### 2.3. The Influence of CAA Interruptions on the Distribution of Hydrogen Bonds Potential Energy

**Figure 8:**
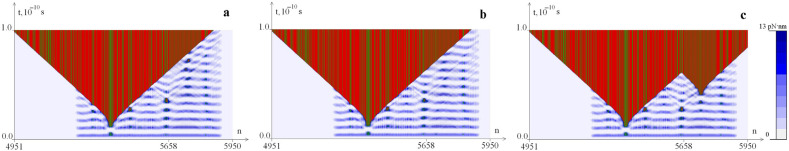
Dynamics of OS zones genesis and hydrogen bond potential energy distribution in base pairs for M_0_ = 8.57 pN·nm with CAA interruptions in the CAG tract: (**a**) corresponds to CAG tract without interruptions, (**b**) corresponds to CAA trinucleotide in the 15th position, and (**c**) to the CAA trinucleotide in the 25th position. The green color in the figure shows the OS in the AT pairs, and the red color shows the OS in the GC pairs. Closed base pairs are highlighted in blue depending on the potential energy values of hydrogen bonds in accordance with the color scale shown on the right.

Figure 8 shows the influence of CAA interruptions in the CAG tract on the genesis of OS zones and the distribution of hydrogen bonds’ potential energy in base pairs. The results are shown for M_0_ = 8.57 pN·nm and CAA interruptions (replacement of the CAG trinucleotide with CAA in positions 15 and 25). It is evident from the figure that CAA interruptions affect the distribution of potential energy and can even lead to the formation of large additional OS zones.

Thus, the probability of the large-sized OS zones genesis on a DNA segment depends not only on the “strength” of the nucleotide sequence of this region but also on the factors that determine the dynamics of DNA.

The obtained results correlate with the data of other authors: the localization of energy in the DNA molecule and the potential energy of pairs of nitrogenous base interactions depend on the parameters of the environment [59,60,61,62]; the viscosity of the liquid surrounding the DNA molecule affects the nature of its wave oscillations [63,64,65,66,67].

In addition, despite the logical assumption that OS zones should appear in DNA regions with a large proportion of AT pairs, since they contain fewer hydrogen bonds than GC base pairs, in regions rich in AT pairs, the probability of the open states genesis will not always be higher [68,69,70].

Thus, the mathematical model allows us to calculate zones of the mechanical energy localization, which is necessary for the formation of OSs, and predict the locations of their nucleation, taking into account the mechanical oscillations of the DNA molecule [61].

The enlarged fragments of Figure 3, Figure 4 and Figure 5 show that the appearance of small OS zones leads to the dissipation of the potential energy of hydrogen bonds in these areas. This can occur due to an increase in the angular velocity of pairs of nitrogenous bases in the OS zones and a more intense dissipation of oscillation energy during interaction with the environment. Therefore, with a stronger torsional effect, localization of potential energy can occur, sufficient for the emergence of a small OS zone at an early stage, as a result of which additional dissipation of energy will occur. This will not allow a large OS zone to form in the future. This explains the non-monotonicity in the OS zones genesis caused by torque.

## 3. Materials and Methods

The study was carried out using mathematical modeling. We used the angular model of the DNA molecule [71,72], which is based on the analogy between a double-stranded DNA molecule and a mechanical system consisting of two chains of interconnected pendulums. In the model, pendulums correspond to nitrogenous bases. This analogy for modeling the rotational motion of DNA bases was first used by Englander et al. [73]. It was concluded that soliton-like solutions of the sine-Gordon equation, with which the authors modeled the rotational movements of the bases, describe the open states of DNA [74,75,76].

In this work, a numerical solution of the mathematical model equations was performed. This makes it possible to take into account the heterogeneity of the DNA molecule, to calculate the probability of the OSs genesis between pairs of complementary nitrogenous bases, and also allow the use of a wide range of external influences. The mathematical formulation of the problem is a system of ordinary differential equations with respect to the angular deviations of the pendulums [77]:

$$I_{1}^{i}\frac{d^{2}\varphi_{1}^{i}(t)}{dt^{2}}=K_{1}^{i}\left[\varphi_{1}^{i-1}(t)-2\varphi_{1}^{i}(t)+\varphi_{1}^{i+1}(t)\right]-$$

(1) $$-\delta^{i}\left(k_{12}^{i}R_{1}^{i}(R_{1}^{i}+R_{2}^{i})sin\varphi_{1}^{i}+k_{12}^{i}R_{1}^{i}R_{2}^{i}sin(\varphi_{1}^{i}-\varphi_{2}^{i})\right)+$$

$$F_{1}^{i}\left(t\right),i=\bar{2,n-1},$$

$$I_{1}^{1}\frac{d^{2}\varphi_{1}^{1}(t)}{dt^{2}}=K_{1}^{1}\left[\varphi_{1}^{2}(t)-\varphi_{1}^{1}(t)\right]-$$

(2) $$-\delta^{i}\left(k_{12}^{1}R_{1}^{1}(R_{1}^{1}+R_{2}^{1})sin\varphi_{1}^{1}+k_{12}^{1}R_{1}^{1}R_{2}^{1}sin(\varphi_{1}^{1}-\varphi_{2}^{1})\right)+F_{1}^{1}(t),$$

$$I_{1}^{n}\frac{d^{2}\varphi_{1}^{n}(t)}{dt^{2}}=K_{1}^{n}\left[\varphi_{1}^{n-1}(t)-\varphi_{1}^{n}(t)\right]-$$

(3) $$-\delta^{i}\left(k_{12}^{n}R_{1}^{n}\left(R_{1}^{n}+R_{2}^{n}\right)sin\varphi_{1}^{n}+k_{12}^{n}R_{1}^{n}R_{2}^{n}\sin\left(\varphi_{1}^{n}-\varphi_{2}^{n}\right)\right)+F_{1}^{n}\left(t\right),$$

$$I_{2}^{i}\frac{d^{2}\varphi_{2}^{i}(t)}{dt^{2}}=K_{2}^{i}\left[\varphi_{2}^{i-1}(t)-2\varphi_{2}^{i}(t)+\varphi_{2}^{i+1}(t)\right]+$$

(4) $$+\delta^{i}\left(k_{12}^{i}R_{2}^{i}\left(R_{1}^{i}+R_{2}^{i}\right)sin\varphi_{2}^{i}-k_{12}^{i}R_{1}^{i}R_{2}^{i}\sin\left(\varphi_{2}^{i}-\varphi_{1}^{i}\right)\right)+$$

$$+F_{2}^{i}(t),i=\bar{2,n-1},$$

$$I_{2}^{1}\frac{d^{2}\varphi_{2}^{1}\left(t\right)}{dt^{2}}=K_{2}^{1}\left[\varphi_{2}^{2}\left(t\right)-\varphi_{2}^{1}\left(t\right)\right]+$$

(5) $$\delta^{i}\left(k_{12}^{1}R_{2}^{1}(R_{1}^{1}+R_{2}^{1})sin\varphi_{2}^{1}1-k_{12}^{1}R_{1}^{1}R_{2}^{1}sin(\varphi_{2}^{1}-\varphi_{1}^{1})\right)+F_{2}^{1}(t),$$

$$I_{2}^{n}\frac{d^{2}\varphi_{2}^{n}(t)}{dt^{2}}=K_{2}^{n}\left[\varphi_{2}^{n-1}(t)-\varphi_{2}^{n}(t)\right]+$$

(6) $$+\delta^{i}\left(k_{12}^{n}R_{2}^{n}(R_{1}^{n}+R_{2}^{n})sin\varphi_{2}^{n}-k_{12}^{n}R_{1}^{n}R_{2}^{n}sin(\varphi_{2}^{n}-\varphi_{1}^{n})\right)+F_{2}^{n}(t).$$

Here,

$\varphi_{j}^{i}(t)$ —angular deviation of the *i*-pendulum of the *j*-chain, counted counterclockwise relatively straight line connecting complementary nitrogenous bases, at time *t*;

$I_{j}^{i}$—moment of inertia of the *i*-pendulum of the *j*-chain;

$R_{j}^{i}$—distance from the center of mass of the *i*-pendulum of the *j*-chain to the thread;

$K_{j}^{i}$—constant characterizing the torque of the *i*-section of the *j*-thread;

$k_{12}^{i}$—constant characterizing the elastic properties of the connection of the *i*-pair of pendulums;

$F_{j}^{i}\left(t\right)$—external influence on the *i*-pendulum of the *j*-chain at time *t*;

n—the number of pairs of pendulums in the system.

In Equations (1)–(6), the first term to the right of the equal sign describes the force action on the *i*-th pendulum from the side of the elastic thread, the second term—from the side of the paired pendulum, the third term—the external force action. The magnitude of the external influence is assumed to be equal to $F_{j}^{i}(t)=-\beta_{j}^{i}\frac{d\varphi_{j}^{i}}{dt}(t)+M^{i}(t)$, where the term $-{\lambda \beta }_{j}^{i}\frac{d\varphi_{j}^{i}}{dt}(t)$ models dissipation effects caused by interaction with the liquid surrounding the DNA molecule, the term $M^{i}(t)$—torque.

By changing the parameter λ, we modeled the change in viscosity of the environment surrounding the DNA molecule.

Equations (1)–(6) make it possible to simulate a hydrogen bond in the *i*-th pair ($\delta^{i}=1$) and break that bond ($\delta^{i}=0$). We assume that a break occurs in *i*-th base pair if the potential energy of hydrogen bonds in this pair exceeds the critical value *E_AT_* for *A*–*T* base pair and *E_GC_* for *G*–*C*; the bond is restored if its potential energy becomes less than the corresponding critical value. The potential energy in the *i*-th pair of nitrogenous bases was taken to be equal to $k_{12}^{i}\cdot l^{2}/2$, where *l* is the distance between complementary nitrogenous bases.

To Equations (1)–(6), we add the initial conditions:

(7) $$\varphi_{1}^{i}(0)=\varphi_{1,0}^{i},\frac{d\varphi_{1}^{i}}{dt}(0)=\varphi_{1,1}^{i},$$

(8) $$\varphi_{2}^{i}\left(0\right)=\varphi_{2,0}^{i},\frac{d\varphi_{2}^{i}}{dt}\left(0\right)=\varphi_{2,1}^{i},i=\bar{1,n}.$$

The values of the coefficients of Equations (1)–(6) are given in Table 1. The data were taken from the work [71].

The values of the energy of hydrogen bond disrupting in AT and GC pairs were taken from [57]: *E_AT_* = 5.1020 pN⋅nm, *E_GC_* = 12.7064 pN⋅nm.

Using the initial conditions (7) and (8), an unperturbed state was specified:

$$\varphi_{1,0}^{i}=\varphi_{1,1}^{i}=\varphi_{2,1}^{i}=0,\varphi_{2,0}^{i}=\pi ,i=\bar{1,n},$$

The torque $M^{i}(t)$ was chosen to be constant in time and spatially localized on the segment [*i*_1_, *i*_2_]:

$$M^{i}\left(t\right)=M_{0}^{i},\;i=\bar{1,n},$$

moreover, *M^i^*_0_ = *M*_0_ at 1 ≤ *i*_1_ ≤ *i* ≤ *i*_2_ ≤ *n* and *M^i^*_0_ = 0 for other *i* values.

The system (1)–(8) was solved numerically.

Calculations and figures were performed using an original computer program written by the authors of the article [78].

## 4. Conclusions

The results of our work allow us a deeper understanding of the mechanisms that lead to open states genesis. The study of hydrogen bonds’ potential energy allows us to predict its redistribution and localization sites depending on the nucleotide sequence and external factors.

The calculations performed showed that hydrogen bonds’ potential energy does not remain constant, and oscillatory movements lead to its redistribution and localization. In this case, open states play an important role, which can effectively dissipate the energy of oscillations. All this allows us to explain the non-monotonic dependence of the probability of open states occurrence with an increase in the value of external torque and a change in the viscosity of the environment.

When studying the occurrence of open states in the *ATXN2* gene, an important role is played by additional OS zones that arise in the CAG tract, in which AT pairs make up only one-third. Studying the processes of nitrogenous bases’ mechanical oscillation potential energy redistribution allows us to answer the question of how the nucleotide sequence in the region of CAG repeats affects the stability of the polyQ tract.

## Figures and Tables

**Table 1:** Coefficients of Equations (1)–(6).

**Type of Base**	A	T	G	C
$I\cdot 10^{-44},kg\cdot m^{2}$	7.61	4.86	8.22	4.11
R, Å	5.80	4.80	5.70	4.70
$K\cdot 10^{-18}$, N·m	2.35	1.61	2.27	1.54
$k_{12}^{H}\cdot 10^{-2},\;N/m$	6.20	6.20	9.60	9.60
$\beta \cdot 10^{-34},N\cdot m\cdot s$	4.25	2.91	4.10	2.79

## Data Availability

The raw data supporting the conclusions of this article will be made available by the authors on request.
